# Development of Tactile Imaging for Underwater Structural Damage Detection

**DOI:** 10.3390/s19183925

**Published:** 2019-09-11

**Authors:** Xi Chen, Gang Wu, Shitong Hou, Jiajun Fan, Ji Dang, Zhiqiang Chen

**Affiliations:** 1 Key Laboratory of Concrete and Prestressed Concrete Structures of the Ministry of Education, Southeast University, Nanjing 210096, Chinaseu_houshitong@163.com (S.H.); jiajun.fan@seu.edu.cn (J.F.); 2 Department of Civil and Environmental Engineering, Saitama University., Saitama 338-8570, Japan; dangji@mail.saitama-u.ac.jp; 3 School of Computing and Engineering, University of Missouri-Kansas City, Kansas City, MO 64110, USA; chenzhiq@umkc.edu

**Keywords:** tactile imaging, underwater structures, damage inspection, nondestructive evaluation

## Abstract

Underwater structural damage inspection has mainly relied on diver-based visual inspection, and emerging technologies include the use of remotely operated vehicles (ROVs) for improved efficiency. With the goal of performing an autonomous and robotic underwater inspection, a novel Tactile Imaging System for Underwater Inspection (TISUE) is designed, prototyped, and tested in this paper. The system has two major components, including the imaging subsystem and the manipulation subsystem. The novelty lies in the imaging subsystem, which consists of an elastomer-enabled contact-based optical sensor with specifically designed artificial lighting. The completed TISUE system, including optical imaging, data storage, display analytics, and a mechanical support subsystem, is further tested in a laboratory experiment. The experiment demonstrates that high-resolution and high-quality images of structural surface damage can be obtained using tactile ‘touch-and-sense’ imaging, even in a turbid water environment. A deep learning-based damage detection framework is developed and trained. The detection results demonstrate the similar detectability of five damage types in the obtained tactile images to images obtained from regular (land-based) structural inspection.

## 1. Introduction

Underwater structures are broadly found and continuously constructed as critical infrastructure systems or components that provide backbone support to modern societies such as other civil infrastructure and structures [1]. Examples include completely or partially submerged structures (e.g., pipelines, dams, and levees) and an extensive inventory of underwater substructures of river crossings or coastal bridges, port structures, offshore wind turbines, and offshore oil platforms [2,3,4,5,6,7]. Due to hazardous loading, long-term environmental deterioration, and man-made (technological) hazards, underwater structures in service are prone to damage. The main damage types in underwater structures may be essentially categorized into the same basic types as they manifest on land-based structures, including cracking and spalling in concrete materials and corrosion and fracture in steel materials. However, unlike damage in land-based structures, the visible appearance of underwater damage is heavily influenced by underwater-specific artifacts and conditions that vary with water flows, the presence of debris or vegetation, fluid–water interactions, lighting, and complex river/ocean-bed terrain [8]. Hence, regular inspection technologies are severely challenged when used to address underwater structures, including the most common visual inspection methods, nondestructive evaluation (NDE) methods, and vibration-based monitoring methods [9,10,11,12].

The most effective approach to underwater-structure inspection to date is diving-based methods that rely on professional divers to perform periodic inspections underwater while carrying lighting and imaging devices (search lights, magnifiers, digital cameras, etc.). The primary advantage of diving-based visual inspection is that trained professionals can thoroughly inspect underwater structures with a great degree of detail when safety permits. When diving-based inspection is applied for underwater structures in shallow water with clear visibility, this method performs well [13]. However, in underwater environments that are either dark, murky, or fast-flowing, structural inspection is more challenging and even life-threatening to divers. If optical imaging devices are used instead, these adverse conditions can significantly influence the imaging quality. Massot-Campos and Oliver-Codina [14] surveyed the optical sensors and methods available for imaging in underwater environments, and found that in situ imaging quality is affected by lighting attenuation, scattering, color absorption, suspended particles, and air bubbles. These conditions lead to blurriness, reduced contrast, and excessive image noise. For river-crossing bridges, this problem is more pronounced, since bridge substructures often sit in rapidly flowing, turbid, and turbulent water [6,15]. In these cases, it is likely that in order to maintain safety, divers cannot effectively locate the structures, but rather often rely on touch-and-sense to identify potential damage. Moreover, for many aged bridges in eutrophic water environments, organic matter often fills the voids of damage, and the divers’ touch-and-sense, although subjective, is indispensable for inspecting the damage [16].

Remotely operational and nondestructive detection (NDE) methods, such as sonar imaging devices, have been studied due to their active capability of penetrating low-visibility turbid water [17,18,19]. Sonar technologies, such as fathometers and advanced two-dimensional/three-dimensional (2D/3D) sonar imaging methods, are efficient in detecting large-scale anomalies such as the riverbed scour around bridge piers. However, they are not effective at detecting small-scale damage (e.g., structural cracks) due to their limited resolution in sonar signals and the possibility that riverbed debris or marine growth may obstruct the signals [20].

Regardless of the use of diver-based optical imaging or nondestructive sonic evaluation, these noncontact inspection methods are operationally challenging, since the operators of these devices need to search objects of interest remotely and inefficiently. To increase the operability for underwater inspection, researchers have explored several remote operated vehicle (ROV)-based platforms that can carry sensors or imaging devices and perform robotic inspection. In recent years, with advances in robotics, ROVs have achieved many desirable properties, including increased endurance, wider/deeper access, semiautonomous navigation, and strong flexibility in underwater activities. To this end, ROVs have been reported to be successful in several cases of underwater inspection [21,22,23,24,25,26,27]. In addition, as intelligent control, machine vision, and artificial intelligence (AI) facilitate automation in robotics, many have proposed the use of autonomous unmanned underwater vehicles (AUUVs) to replace ROVs for underwater inspection [28,29,30,31,32]. These AUUVs can be further equipped with a robotic arm and sensing payloads to realize opportunistic and intelligent sensing, as a trained professional does (e.g., touch-and-sense). This transformation motivates the authors of this paper to reflect on the robustness and thoroughness of diver-based visual inspection, and to further envision the possibility of developing robotic systems that can approach human diver efficiency and accuracy for underwater structural inspection.

Biologically inspired by human-based touch-and-sense, the authors in this paper aim to develop a robotic tactile imaging and real-time computing system, which can be integrated with an AUUV in the near future for fully autonomous underwater structural inspection, including damage localization, identification, and quantitative assessment. The rationale is briefly outlined as follows. First, optical imaging and its enabled machine-vision methods provide intuitive measurements in terms of spectral intensities that manifest as shapes and other patterns. Compared with other types of NDE imaging (e.g., sonar imaging), optical imaging can yield high-resolution imagery data and can realize structural damage detection at necessary scales. However, optical imaging relies on photons transmitted in transparent media (e.g., air, water, and glass) when performed remotely, hence suffering from limitations in ambient illumination and occlusion. On the other hand, tactile perception, as a biological mechanism that is an advanced capability only possessed by animals and human beings, can perceive surface roughness, patterns, and shapes that transmit to neural systems for high-level understanding. Many biologically inspired tactile sensors have been proposed (as reviewed later). Yuan et al. and Li et al. developed a tactile imaging device using a polydimethylsiloxane (PDMS)-based elastomer called GelSight [33,34,35,36,37]. This mechanical elastomer-based imaging device has been used in sensing texture patterns, measuring field pressure, and enabling 3D surface reconstruction. To this end, PDMS-based tactile imaging has not been developed or tested for underwater inspection. Another interesting property of PDMS is its super hydrophobicity, which eliminates the appearance of water artifacts (e.g., air voids or bubbles) on the PDMS–water contact surface, enabling high-quality imaging [38,39]. By combining the merits of optical imaging and tactile sensing, a highly efficient, precise, and robust inspection technology may be possible, as will be investigated in this paper.

In this paper, an elastomer-based Tactile Imaging System for Underwater Inspection (TISUE) system is developed and tested as the first stage of the aforementioned vision for robotic underwater structural inspection. This system first overcomes the limitations of existing remote optical or sonar-based NDE solutions. This imaging mechanism relies on the deformation of a high-viscoelastic material (elastomer) to initiate surface touching that creates a geometric molding of the microscopic structures on the surfaces of underwater objects. Further, by exploiting the optical transparence of the elastomers, a novel lighting apparatus is designed for contact-based optical imaging. To prototype this novel tactile imaging method, a mechanical support is designed for robotic underwater inspection. Finally, to realize the notion of real-time situational awareness, an intelligent data communication and image processing pipeline is developed and further tested in this paper. A laboratory-based experiment is specifically developed in this paper for testing and evaluating the performance of the system, which demonstrates the feasibility of the proposed tactile imaging for underwater damage inspection. Although the scientific principle is similar to that adopted in previous efforts [35,36,37,38,39], four important differences are highlighted:

(1) Our efforts mainly rely on the optical transparency and hydrophobicity of PDMS in the contact process for transmitting reflected light intensity to create images. GelSight adds a layer of coating on the surface of PDMS in contact with objects, which facilitates 3D imaging.
(2) In accommodating the difference in optical media in generating images, our system employs white light-emitting-diode (LED) strips with an inclined illumination angle to enable optical transmission and reflectance prior to imaging. GelSight employs LED arrays of different colors to illuminate the elastomer surface with a tilted angle of 71° for the purpose of 3D imaging.
(3) GelSight acts solely as an imaging device. Our developed system integrates tactile imaging with embedded data transmission, real-time display, and potentially real-time image-based processing.
(4) Last, our system is specifically developed for underwater structural-damage inspection for largely 2D surfaces with structural anomalies that have microscopic depths (e.g., surface cracks or texture-like spalling). Therefore, a 3D reconstruction of surface shapes is not critical. However, it is our understanding that GelSight is optimized to perform 3D surface reconstruction.

## 2. Related Work in Tactile Theory and Underwater Image Restoration Techniques

There are many kinds of tactile sensors, including piezoresistive tactile sensors, piezoelectric tactile sensors, capacitive tactile sensors, and other types of tactile sensors. A piezoresistive material will change its own resistance when it is subjected to external pressure. By measuring the change of resistance, the external pressure can be measured. Therefore, a piezoresistive material can be used as the pressure-sensitive material of a tactile sensor to detect the tactile force. In 2012, Tee et al. [40] mixed nickel particles with nano-microstructures with supramolecular polymers to produce piezoresistive materials, which were used in the design of tactile sensors. The developed sensor showed good tactile force detection performance. Piezoelectric materials generate electric charges when they are subjected to external pressure, and the external force can be detected by measuring the amount of charge generated. PVDF (polyvinylidene fluoride) has piezoelectric properties after polarization treatment, and can be used as a pressure-sensitive material for tactile sensors. In 2001, Qasaimeh et al. [41] integrated PVDF into the sensitive element of a tactile sensor array and measured the contact force by using the piezoelectric characteristics of PVDF. The structure of a capacitor usually consists of two capacitor plates and a dielectric layer. Under the action of external force, the dielectric layer is compressed, which makes the distance between the two capacitor plates change, and thus the capacitance value changes. The capacitive tactile sensor array with a flat electrode as the capacitive electrode plate has simple structural characteristics, and it is easy to measure the tactile force. In 2008, Pritchard et al. [42] designed a thin layer of poly (paraxylene) between two flat electrodes as a dielectric layer to produce a tactile sensor array of 10 × 10.

It is noted that the notion of tactile imaging is different from the general notion of tactile sensing. In general, tactile sensing devices imitate biological cutaneous receptors to measure physical properties at surface points, such as pressure or temperature, and have been an active topic for designing robotic sensing [43,44,45]. More than merely point-based measurement, tactile imaging translates field measurements into two-dimensional or three-dimensional (2D or 3D) digital data as images. To this end, different tactile imaging mechanisms are designed, most of which convert mechanical pressure into images (hence called mechanical imaging, alternatively) [46]. Many mechanical image devices exist and have been applied extensively in medical practices [47,48,49].

To translate touch-enabled sensory information into imagery data, the use of an elastomer has been actively explored. The basic principle is that by using an elastomer as the touching interface against an object, the elastomer conforms to the surface shape of the object when it is pressed. Then, this molded shape is optically imaged due to the transparency of the elastomer. Elastomers usually refer to viscoelastic polymer materials that have a low Young’s modulus and a very high failure strain. As a high molecular-weight silicone compound, polydimethylsiloxane (PDMS) is a popular elastomer that is often used to fabricate various microdevices because of its outstanding physical and chemical properties, such as optical transparency, elastic flexibility, biocompatibility, nontoxicity, and nonflammability [50,51]. Hard (nonliquid) PDMS can be prepared using different mixing ratios of curing agents to prepolymer to obtain cross-linked solid PDMS that can be stretched, bent, and compressed in any direction [39,52], hence ready for the development of an elastomer ‘mold’. A PDMS-based elastomer, once pressed to any solid surface, can conform to the surface shapes at tunable geometric scales, including the microscopic texture details of the surface.

Traditional image enhancement methods are divided into spatial domain and frequency domain. The method of spatial domain enhancement is to directly process the pixels of the image. Based on gray-scale mapping, the corresponding mapping transformation is selected according to the image characteristics and different enhancement purposes. Frequency domain enhancement is an indirect image processing method. It performs some filtering processing on the transformed coefficients in a certain transformation domain of the image, and then inversely transforms them to the spatial domain to achieve the enhanced image. According to the characteristics of underwater images, in recent years, scholars have proposed many typical underwater image enhancement methods, for example, based on the quaternion attenuation coefficient inversion recovery processing algorithm [53], integration, RGB and HSI color model enhancement algorithm [54], color correction method based on the ACE model [55] point spread function (PSF), processing algorithm [56], underwater image based on wavelength compensation to “atomization” enhancement methods [57], etc. Some other methods mainly improve the underwater image enhancement algorithm, such as the underwater image enhancement method based on RETINEX [58].

## 3. Tactile Imaging Design for Underwater Inspection

### 3.1. Concept Design and Advantages

**Figure 1:**
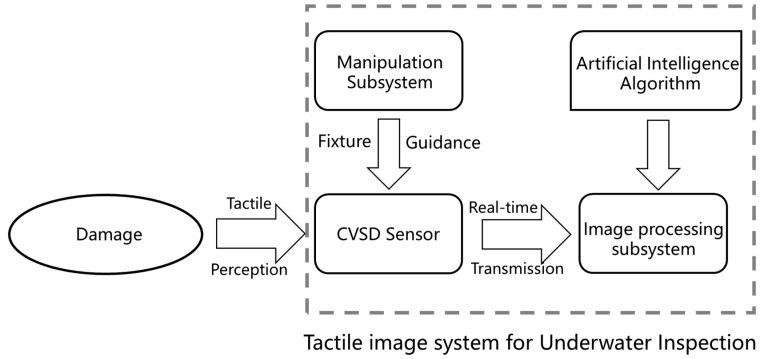
Concept design of the tactile imaging system for underwater inspection (TISUE).

Addressing the challenges of structural damage detection for underwater structures and the limitation of existing nondestructive methods, in this paper, we conceptualize, prototype, and test the Tactile Imaging System for Underwater Inspection (TISUE). Figure 1 provides the schematics of the TISUE system. As shown in Figure 1, the system has two major components, including the imaging subsystem and the manipulation subsystem. The imaging subsystem, including image acquisition, storage, and display analytics, is designed and prototyped into one device, termed the contact-visual sensing device (CVSD). To ensure the operability of the CVSD in rapid and deep water, a mechanical manipulation and control device is developed to carry the front end of the CVSD and control its position. Details about the design of both devices are outlined in Section 3.2 and Section 3.3, respectively. Figure 1 illustrates the concept design and operation of the proposed TISUE system. As depicted in Figure 1, with the aid of a mechanical fixture and guiding support system, one professional can operate the TISUE system to conduct an underwater inspection. This illustration conveys the most immediate use of the proposed system; nonetheless, as proposed earlier in this paper, a robotic arm-based TISUE system is envisioned that integrates with an AUUV system with enhanced automation.

The CVSD is designed to capture the structural surface of underwater objects, and then, the images are stored in a built-in SD card pending further processing. To enable real-time observation and operation, a mobile tablet-based display is connected with wired real-time image transmission. While imaging, the images can be displayed for real-time visualization; potentially, real-time imaging processing can be conducted by exploiting the embedded computing capability of the mobile device. In this paper, we propose to use deep learning-based methods to process the images to test the detectability of structural damage in the obtained tactile images. In the future, as a large number of underwater tactile images are archived and semantically labeled, more generalized deep-learning algorithms can be implemented.

### 3.2. Optical Imaging and Illumination Design

**Figure 2:**
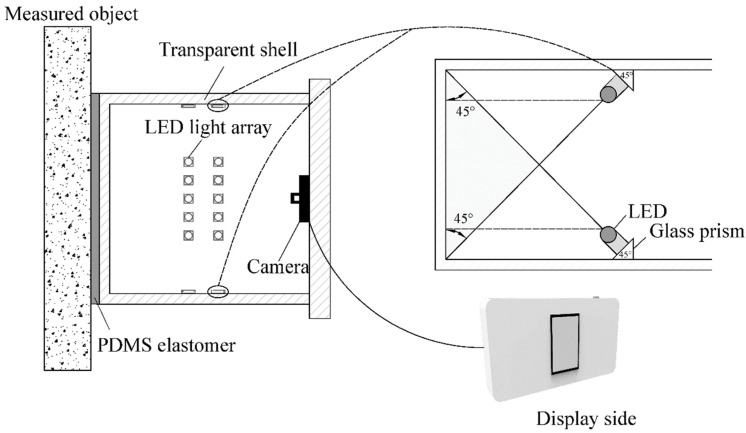
Imaging front-end of the contact-visual sensing device.

In this paper, a contact-visual sensor, which is at the front end of the CVSD device, is designed and fabricated (Figure 2). The sensor takes advantage of a flexible, transparent, and hydrophobic material—PDMS—to actively touch the structural surface, conform to the surface’s microscopic details, and take pictures with a high-resolution camera.

To achieve the operational stiffness of the PDMS material, a series of mixing testing was conducted to determine the optimal curing scheme [59,60,61,62]. It was concluded that a ratio of 1:30 (between the curing agent and elastomer base) empirically demonstrates the optimistic detectability for concrete surface damage. The shape of the PDMS is square (20 mm × 20 mm), with an even thickness of 4 mm. A plexiglass (15-mm thickness) plate is used as the base for the transparent PDMS media. In the determination of elastomer thickness, previous studies have noted the mechanical properties of PDMS in various thicknesses [63,64,65,66]. As identified by Wang and Volinsky [67], the elastic modulus of PDMS is approximately 0.56 MPa. However, the PDMS would be easily cut by uneven or angular concrete surfaces during the contact process if the PDMS was too thin. Thus, before the underwater experiment (Section 4), we selected 4-mm PDMS after several trial-and-error attempts.

The illumination field inside the device is the crucial factor enabling optical imaging and affecting the image quality. Most artificial light sources often create highly nonuniform lighting in the target scene. Furthermore, if the lighting sources are not placed appropriately with reflective materials in the field, specular reflections can occur that mask the details of the target and create more unevenly illuminated spots. To create a uniform, bright, and transparent environment inside the device, four LED strips that are obliquely 45 degrees to the sidewall are attached to the side of the casing. With this design (Figure 2), the range of LED lights can be used to a greater extent to form an imaging environment with parallel light sources. For color selection, to ensure that the actual conditions on the surface of the measured subject are completely recorded in images, a white color (390–760 nm), which includes all the wavelengths of visible light, should be adopted. Based on its transmission curve [68], the LED light can transmit to the PDMS and reflect back to the camera without significant spectral modification.

When prototyping the sensor, to achieve excellent water-pressure resistance and waterproof performance, the sealing of the device needs to be ensured with high-strength stainless steel bolts and coated with a layer of waterproofing. In addition, a rubber ring is also arranged between the casing and the cover plate to enhance the overall waterproof performance of the device.

**Figure 3:**
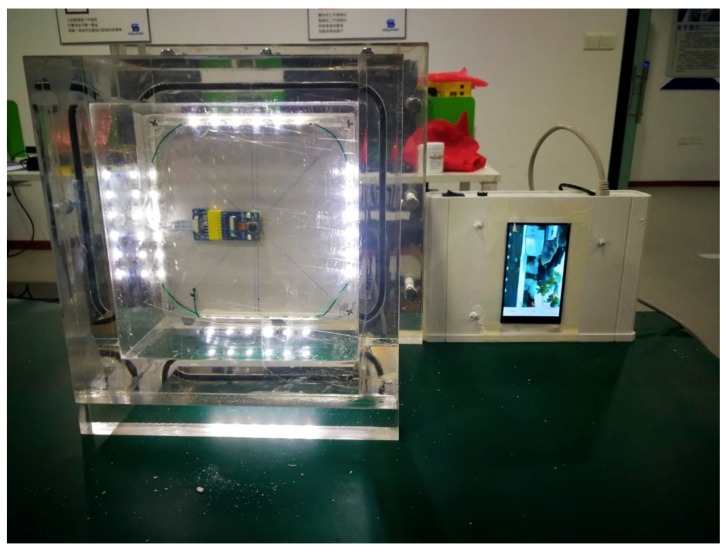
Prototype of the contact-visual sensing device (CVSD): the front-end optical sensor and the real-time display.

Figure 3 displays the realized prototype sensor at a working state with wired connection to a display device. As shown in Figure 3, the image acquisition device is housed in a cubic-type plexiglass box with its front-end attached to the PDMS interface and the inner back surface attached to the imaging device. The inner sidewalls are installed with inclined LED strips.

### 3.3. Electronics for Imaging, Data Transmission, and Display

**Figure 4:**
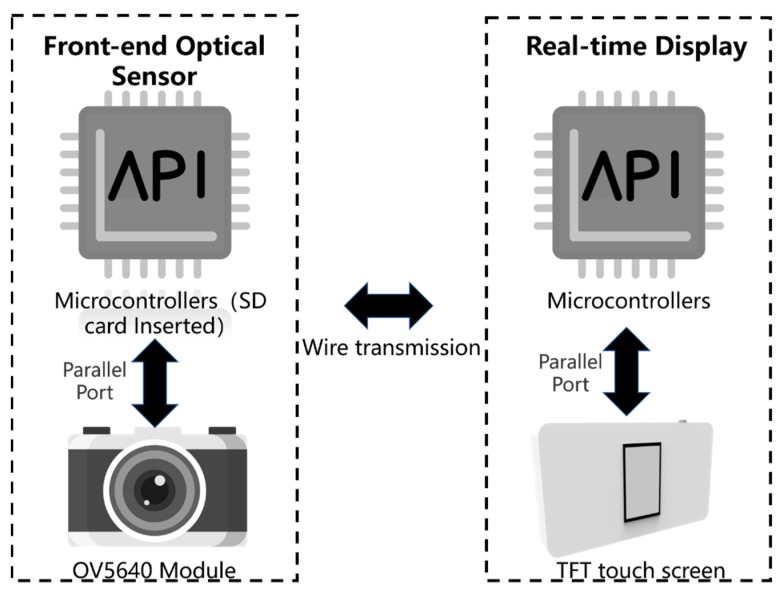
Systematic design of imaging and data communication.

Figure 4 depicts the electronic design for the imaging system. A high-resolution imaging device (OV5640 module) is placed at the center of the end side of the imaging case. The image processing subsystem is configured to perform batch processing on the image acquired by the high-resolution imaging device and output the processing result.

**Figure 5:**
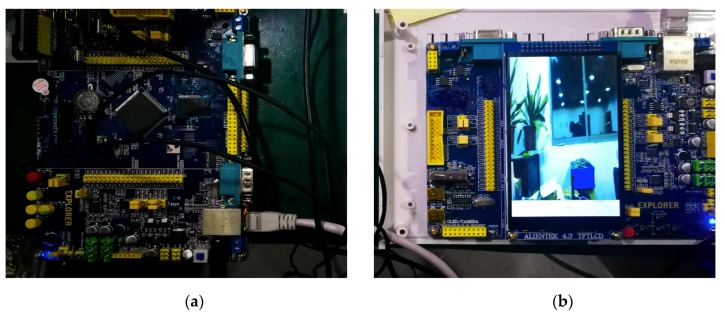
Interior electronic realization: (**a**) data transmission; and (**b**) visual display.

Specifically, the high-resolution camera in the imaging device is placed directly below the elastomer. The image acquisition subsystem uses two STM32F4 series MCUs as the master port, all of which communicate through the data parallel port A; one is connected to the camera, and the other is connected to a 4.7-inch TFT touch screen. The camera is connected by the network cable B; the camera adopts the OV5640 module with a resolution of 5 million pixels. It also owns an interface for secondary development, which can realize real-time capture automatically. The communication mode between the camera and display is set to a custom mode. The FSMC mode is used to connect the screen with the parallel port. The display subsystem additionally includes a network interface for communicating with the image acquisition subsystem to accept image information. Details of the entire systematic design of the device are shown in Figure 5.

To ensure data transmission in the harsh underwater environment, we chose wired transmission rather than a wireless mode (which is possible for land-based imaging and data transmission). Finally, the acquisition frequency of the entire image acquisition module is set at 3 s per frame (or 1/3 frame/s, which can be adjusted to a higher frame rate). In this paper, this slow frame rate is selected, considering the possibility of obtaining images that carry sufficient lighting differences from turbulent water flow at the same location. All the images captured are stored in the built-in SD card in the JPG format for visualization and image processing.

### 3.4. Manipulation Subsystem Design and Prototyping

**Figure 6:**
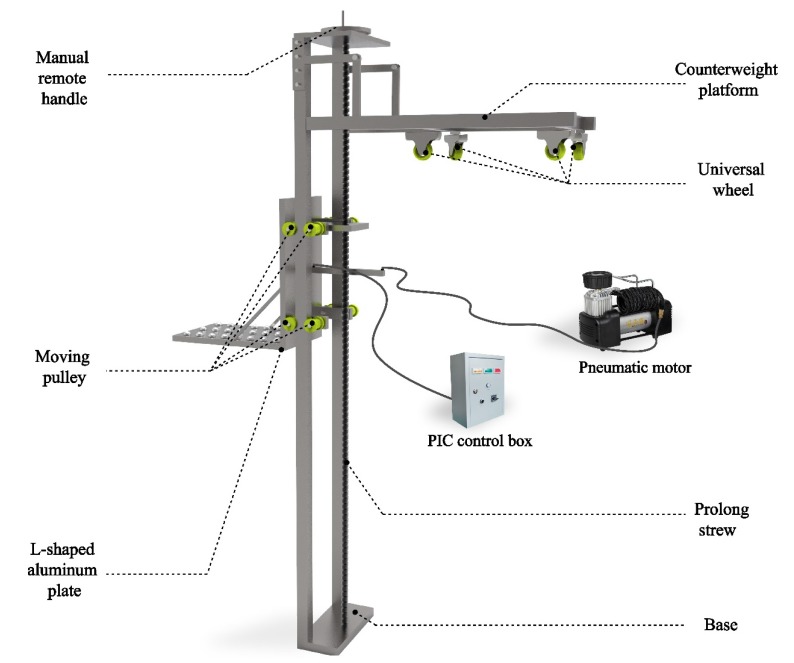
Details of the mechanical device.

To make the sensor perform stably, continuously, and efficiently in an underwater environment, a mechanical device for carrying the sensor is also designed (Figure 6). The apparatus includes the inspection module, a vertical control module for controlling the vertical height of the inspection device, and a traverse pneumatic module for controlling the horizontal positioning of the detection device. The vertical control module and the lateral pneumatic module are connected. The inspection module comprises a depth sensor and a pressure sensor; the depth sensor is used for sensing the specific orientation of the underwater inspection device, and the pressure sensor is used for capturing the force feedback generated by the contact between the inspection device and the measuring object. An inflator timely delivers high-pressure gas to the pneumatic module in a mechanical device and a PIC control box to keep the CVSD forward actively along the track of the L-type aluminum plate with the help of the inflator during each inspection task. This device requires only a small amount of manual operation to complete the inspection work from several meters to several tens of meters in underwater conditions, and can also realize long-distance control operations. In addition, the disassembly or assembly of the apparatus is convenient, and all the devices can be reused, which greatly saves economic costs and improves the efficiency of inspection.

The working principle of the present invention is as follows: by rotating the manual crank, the long lead screw is driven to rotate, thereby driving the detecting device and the L-shaped plate to move up and down. The specific working mode of the transverse pneumatic module occurs when the switch of the PIC control box is turned on; the air pump intake, which is connected to PIC, and the pneumatic expansion device together drive the detecting device to move forward until it contacts the measured object. The counterweight module is made up of a counterweight platform and four universal wheels. This module mainly utilizes the principle of leverage to achieve the balance and stability of the entire device by adding a counterweight on the platform.

## 4. Performance Evaluation

### 4.1. Experimental Design

**Figure 7:**
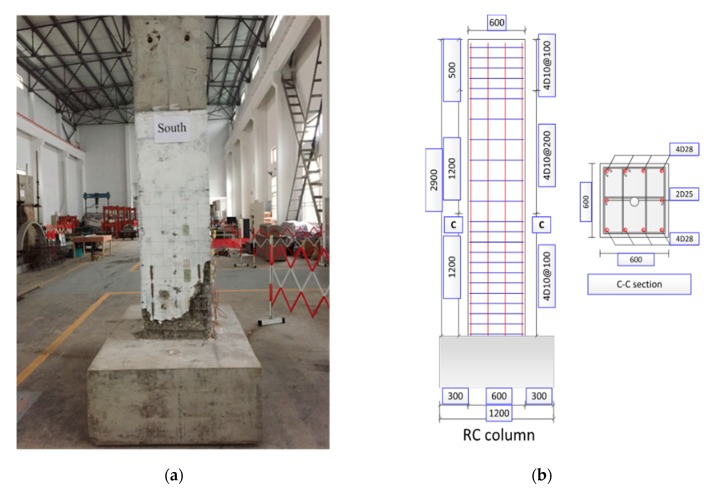
Concrete column configuration: (**a**) loaded structure with damage patterns; (**b**) original reinforcement details.

To verify the feasibility of the proposed concept and the tactile imaging system prototype, a reinforced concrete column was prepared and tested in the laboratory (Figure 7). The column is a full-scale reinforced concrete cast-in-place square-section column. Before underwater inspection, this column was loaded laterally with a low-cycle procedure. At the ultimate failure state, a large number of different types of damage were observed on the surface of the column, including cracks, defects, spalling, etc. It was noted that the introduced damage herein was not completely equivalent to those found in real bridge piers, and the damage types in terms of their morphological shapes are similar to underwater damage.

**Figure 8:**
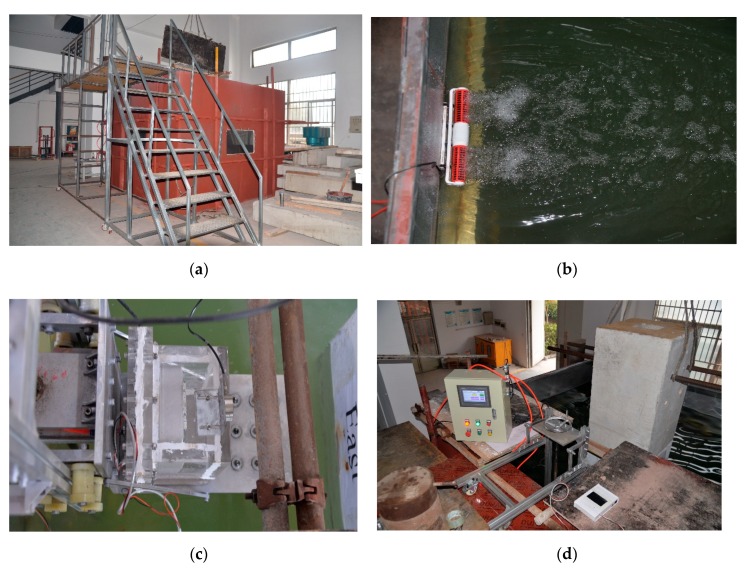
Overview of underwater inspection: (**a**) Water tank; (**b**) flow/turbulence generator; (**c**) tactile imaging device in water; (**d**) control, visualization, and mechanical supports.

To construct an underwater experimental environment, a 3 × 3 × 2.5 m iron tank is manufactured in the laboratory. To simulate the realistic underwater environment, a turbulence generator is installed on the sidewall of the tank to create a turbulent flow. In this experiment, we maintained an average flow velocity of 2.5 m/s in the tank. To achieve a low-visibility condition, sufficient sludge and sewage are added to the tank and measured by a turbidity meter. In this experiment, in addition to the clear-water condition, a low visibility of 400 NTU (or nephelometric turbidity units) condition is created. Figure 8a,b show the water tank from different perspectives.

Prior to the inspection, the mechanical support was operated to transport the imaging device underwater, and an ‘initiation’ command was issued via the PIC that ensures the device is in contact with the surface of the column (Figure 8c). When the device is turned on, the display system will provide the surface condition of the current measurement area in real time (Figure 8d). When damage is found, the device can be kept in a stable and static state, and the pneumatic module is correspondingly opened to apply a certain amount of force to the device. In this experiment, a nominal force of 1500 N measured by the load cell was used. As such, the elastomer at the front end of the device is in full contact with the surface of the column. In our experiment, it was observed that such tight contact substantially eliminates the possible impact of in situ disordered water flow on the image quality.

A total of five different types of damage were collected. Details of their underwater location, type, applied force, and number of captured images are listed in Table 1. It is worth noting that at each damage scene, a large number of images (5000) were captured. These images at each scene essentially have the same field of view. However, as originally designed in this experiment, to realistically simulate the underwater environment, an artificial flow generator is introduced into the water tank that randomly creates different flow patterns at each imaging instant, which in fact introduces different refracted light into the chamber of the transparent imaging device (as shown in Figure 3). In the meantime, despite the use of a nominal pressing force of 1500 N, the pressure at the contact surface is not even and varies in a range, as the mechanical arm is influenced by the water flow. Therefore, the water voids and bubbles appear different at the corners of the PDMS contact area. By setting a slow frame rate (one frame per three seconds), each captured image, although showing the same object in the foreground, differs in background illumination and water artifacts. This information is exploited in the image processing phase, considering the use of a deep learning framework.

### 4.2. Sample Images and Descriptions

**Figure 9:**
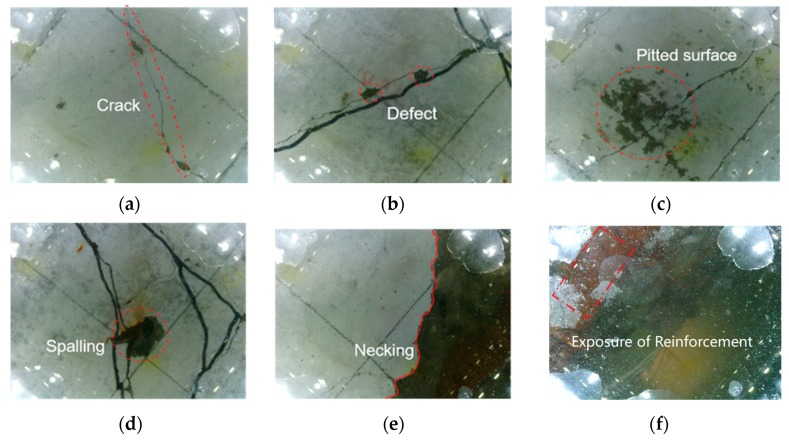
Sample tactile images for the experimental underwater structures: (**a**) Crack; (**b**) surface defect with small voids; (**c**) surface defect with pitted voids; (**d**) spalling; (**e**) necking; (**f**) exposed reinforcement.

Figure 9a–f show selected images acquired by the CVSD. Figure 9a displays surface cracks in the concrete; Figure 9b,c display minor surface defects showing small voids and pitted voids, respectively; Figure 9d displays the spalling of local concrete; Figure 9e displays the necking of the concrete column; Figure 9f displays an exposed and corroded steel bar due to massive concrete spalling.

To further highlight the advantages of picture sharpness captured by CVSD, several controlled trials were conducted. Figure 9 displays four image samples at different turbidity levels with CVSD and without touching. Figure 9a is a crack image obtained by a noncontact inspection method (400 mm from the column surface) under a water turbidity of 300 NTU; Figure 9b is a crack image obtained by a noncontact inspection method (400 mm from the column surface) under a water turbidity of 400 NTU; Figure 9c is a crack image obtained by CVSD under a water turbidity of 300 NTU; Figure 9d is a crack image obtained by CVSD under a water turbidity of 400 NTU.

**Figure 10:**
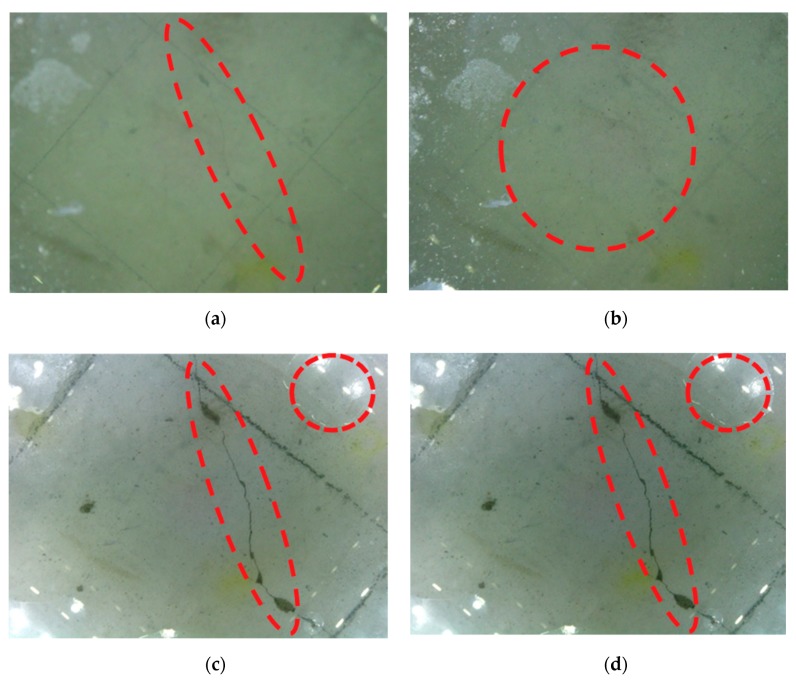
Images samples at different turbidity levels with CVSD and without touching. (**a**) Crack image (300 NTU, 400 mm from the column surface); (**b**) crack image (400 NTU, 400 mm from the column surface); (**c**) crack image (300 NTU, acquired by CVSD); (**d**) crack image (400 NTU, acquired by CVSD). NTU: nephelometric turbidity units.

Figure 10 shows the advantage of tactile imaging via CVSD. In terms of the image quality and the richness in damage patterns captured by the images, the contact-based images are superior to those detected by the noncontact images, although the same CVSD was used. From Figure 10a and b, to the naked eye, it can be seen that with the increase of turbidity, the damage information that can be provided in the picture significantly decreases. Under the condition of 300 NTU, the damage on the surface of the column is faintly visible. When the turbidity increases to 400 NTU, the damage on the surface of the column cannot be seen in the images collected by the noncontact capturing at the same acquisition distance.

Meanwhile, Figure 10c,d each show round air voids (circled in red) at the corners, because the elastic body is pressed during contact with the measured column, while residual water in the original corresponding area is sent to the corner of the field of view. This effect not only provides excellent conditions for CVSD to collect damaged images, but also demonstrates the unique transparency and hydrophobicity of PDMS materials. In addition, it can be certain that the turbidity has little effect on the quality of images collected by the CVSD. This phenomenon verifies one advantage of achieving a fine imaging environment by internal means to eliminate the limitations of natural conditions in CVSD.

### 4.3. Image-Based Processing for Damage Detection

A deep learning (DL)-based algorithm is proposed in our work by modifying the fully connected convolution neural network (FC-CNN or simply FCN) proposed in [69,70]. To adapt to underwater structural damage detection, several modifications (such as redefined loss functions) were introduced. The algorithm related to the FCN model mainly includes three main goals: image classification, object localization, and semantic segmentation. Image classification is made up of making a prediction for a whole input, i.e., predicting the object in an image or even providing a ranked list if there are many objects. Not only the classes but also additional information regarding the spatial location of those classes, e.g., centroids or bounding boxes, are provided by object localization. Semantic segmentation makes dense prediction inferring labels for every pixel that is labeled with the class of its enclosing object or region. Following the steps of image classification, object localization, and semantic segmentation, the evolution of defect recognition progresses from coarse-grained to fine-grained inference.

**Figure 11:**
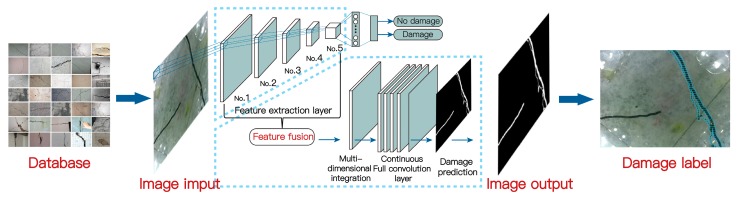
Methodology workflow of the proposed framework.

In this section, an architecture of FCN that combines semantic information from deep, coarse layers and appearance information from shallow, fine layers to produce accurate and detailed segmentation will be built for underwater damage, i.e., cracks and defects. Figure 11 displays the methodology workflow of the proposed framework. The FCN network is trained with a set of 20,000 manually labeled images (via manual semantic segmentation). The details for implementation are not a focus of this study due to the limited length of this paper. Specifically, the images used as shown in Table 1 have only five different scenes. As mentioned earlier, to increase the scene complexity, 5000 images were collected at each scene. At each imaging instance, the lighting condition and the appearance of water artifacts were different in each image due to in situ refraction and reflection as a result of water flow and variation in the contact pressure. Therefore, the resulting DL model solely fits into this imagery data set, which learns not only the underlying five damage scenes, but also the complex imagery noises (more remarks are provided later). All of the computation is implemented via a desktop computer with an Intel i5-8400@3.60 Hz CPU core and eight core processors, 16 GB RAM, an NVIDIA GTX-1060 GPU with 8 G of memory, and a 64-bit system type. Caffe, a noted DL framework, is used to perform this calculation [71].

**Figure 12:**
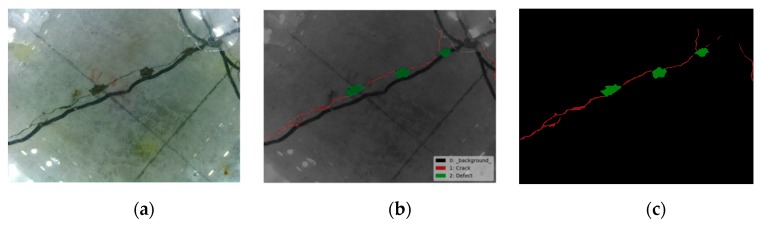
Recognition results for crack images. (**a**) Raw image; (**b**) labels of damage type; (**c**) recognition results.

**Figure 13:**
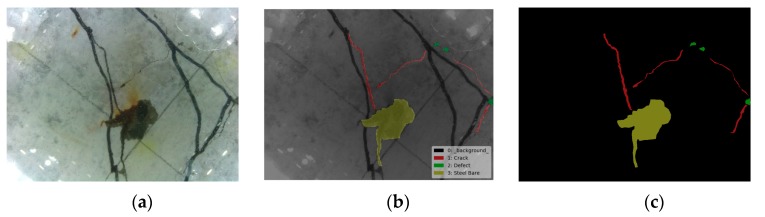
Recognition results for defect images. (**a**) Raw image; (**b**) labels of damage type; (**c**) recognition results.

**Figure 14:**
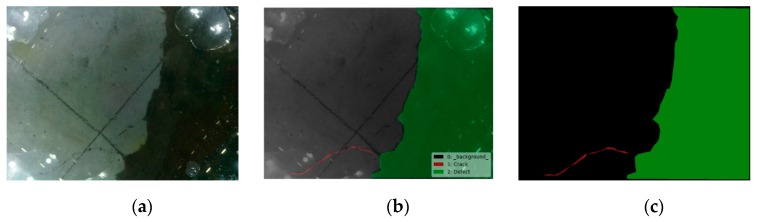
Recognition results for necking images. (**a**) Raw image; (**b**) labels of damage type; (**c**) recognition results.

The semantic object detection and segmentation results are shown in Figure 12, Figure 13 and Figure 14 using the trained FCN model. From these figures, it is not difficult to find that the results identified by this algorithm are very close to the original distribution and details of damage. Whether inspecting macroscopic damage, such as void-like defects or necking, or microscopic damage, such as cracks, CVSD can acquire high-definition damage images in extreme environments such as those with low visibility and rapid current flow. The FCN model, when employed as a semantic damage detector, shows the detectability of these tactile images. We can conclude that with specific underwater artifacts and artificial lighting, the TISUE system as a whole and the CVSD device specifically function as expected.

### 4.4. Remarks and Discussion

In the experiment, a total of five types of underwater damage were captured using the TISUE system and further detected in a deep-learning program. The five damage types, including surface cracks, void-like defects, corrosion, necking, and exposed reinforcement, cover nearly all types of typical damage for underwater structures. From images collected by the device, the contact visual sensor device proposed in this paper can effectively overcome the influence of an underwater low-visibility and unstable water environment on image quality and acquire high-definition images, and thus fundamentally guarantee ensuing image processing and advanced damage identification. The acquisition of high-definition images mainly benefits from novel tactile imaging technology and system implementation in this paper. Herein, we focus on the central values and issues in the experimental process.

In Section 3, we mentioned that the primary technical means of “contact visual sensing” is to collect images of potential damage for underwater structures. Due to the combination of visual objectivity and tactile realism, this concept has substantial advantages and competitiveness in the detailed description and accurate identification of local damage. The use of the PDMS material as the medium to acquire contact-based structural surface topology and the tactic configuration of LED-based lighting are the keys to this success. However, to this end, we note that a computational mechanics-based analysis should be developed considering the complex mechanical and flow–structure boundary conditions. As such, a quantitative guideline can be defined for the accurate operation of the tactile imaging device. This need is being addressed in our ongoing work.

To make the test conditions closer to realistic underwater conditions, we simulated the water flow at 2.5 m/s and the water turbidity at 400 NTU. During the test, ensuring the stability of the performance of the contact-based sensing device was a challenge. Thus, a mechanical device that carries the pneumatic module to transport the sensor to a select location was designed, which provides the basis for applying the pressing force to the sensor to achieve a working contact pressure. Judging from the effects of images collected by the device, the mechanical support subsystem is very useful in solving the “jitter” of the device caused by the water flow impact. It is worth mentioning that this mechanical system is developed for manual operation provided that a trained engineer operates the system. Robotic arm-based development and integration with an AUUV system are pending for future development.

Previously, because of the limitation in the paucity of underwater images and the poor quality of these images, underwater image processing has been mostly treated case-by-case, and mainly using low-level image processing methods. It is envisaged that with the innovation in this paper, underwater damage detection can enable the notion of big data in terms of underwater structural damage images. This will give rise to the opportunity to perform the advanced image-based modeling of underwater structures with high-resolution information and high-definition condition modeling and assessment.

The deep-learning algorithm used in this paper provides an example of the accurate identification of underwater damage. The DL framework aims to illustrate the detectability of damage in the obtained tactile images. This means that one should not generalize it to other images acquired in different imaging environments. In other words, analogous to what an edge detector does for edge detection, herein it is used as a semantic object detector that identifies the damage type and outputs the contours. Nonetheless, the use of the DL framework provides the promise of generalization when underwater images from a large number of scenes (large underwater images) are obtained.

Finally, some specific future efforts are suggested herein. First, the test object in this paper is a square-section concrete pier substructure. Structures of different functions, materials, and shapes should be tested in controlled underwater environments with sufficient similarity to realistic situations. Second, the existing CVSD has a fixed (narrow) field of view (FoV); wider FoVs should be attempted. Third, the preparation of the elastomer material uses a 50:1 ratio of silica gel and curing agent. Optimal elastomer design and testing are necessary. Finally, we believe that the concept of tactile imaging for structural damage detection may be extended to low-visibility, complex, and harsh environments. Specific ad hoc research for using the CVSD concept for other extreme environments is worthy of exploration.

## 5. Conclusions

Underwater structural-damage inspection has been a challenging engineering problem and mainly relies on diver-based visual inspection. Emerging technologies include the use of remotely operated vehicles (ROVs) for improved efficiency. However, they cannot provide the accuracy and quality that a trained professional diver can provide. The existing nondestructive technologies (e.g., soar-based sensing) lack efficiency when the location of potential damage is beyond records or engineering judgments. Inspired by the biological sensing of human-based touch-and-sensing and further motivated by performing an autonomous and robotic underwater inspection, a novel Tactile Imaging System for Underwater Inspection (TISUE) is designed, prototyped, and tested in this paper. The system has two major components, including an imaging subsystem and a mechanical operation subsystem.

The proposed core concept of tactile imaging can efficiently and accurately detect underwater structural damage, even in a low-visibility underwater environment. Without any attachment on the surface of underwater structures, the tactile sensor can acquire large amounts of images at high definition. By utilizing this novel method, the impact of low visibility and turbulent water flow on image quality can be effectively diminished. The following advantages of the imaging system are confirmed by the system prototyping and the experimental evaluation in this paper:

(1) The imaging subsystem is able to provide a refined characterization of local damage on the surface of the object through the transparent flexible PDMS material. The PDMS as an elastomer is the right material for conforming to the structural surface with microscopic damage details from a variety of common damage types.
(2) With little manual operation, the operation subsystem can semi-automatically realize underwater inspection and not be disturbed by extreme measuring environments.
(3) Through a deep learning-based framework that realizes automatic semantic damage detection, the obtained underwater damage is fully detectable with semantic labeling of both types and contours. This implies the possibility of conducting accurate quantification if pursued in the next step.

Several limitations and future directions are noted in this paper (Section 4.4). With the findings above, the system proposed in this paper can be further developed to be ready for practical adoption. More advanced integration with autonomous systems may genuinely transform the practice of structural damage inspection for the inventory of many types of underwater structures in the future.

## Figures and Tables

**Table 1:** Summary of collected damage image information.

Types of Damage	Location	Depth of Water (m)	Number of Images Collected
Crack	North	1.23	5000
Steel Corrosion	North	1.74	5000
Concrete Defects (small voids and pitted surfaces)	South	1.42	5000
Necking	East	1.55	5000
Exposure of Reinforcement	West	1.65	5000
